# Supplementary material for: Climate change rapidly warms and acidifies Australian estuaries
Source: Nat Commun. 2020 Apr 14;11:1803. doi: 10.1038/s41467-020-15550-z (PMC7156424; doi:10.1038/s41467-020-15550-z)
Supplement: Supplementary file 1 — Supplementary Information [file 41467_2020_15550_MOESM1_ESM.pdf]

Supplementary Information for:

Climate change rapidly warms and acidifies Australian estuaries

Scanes et al.

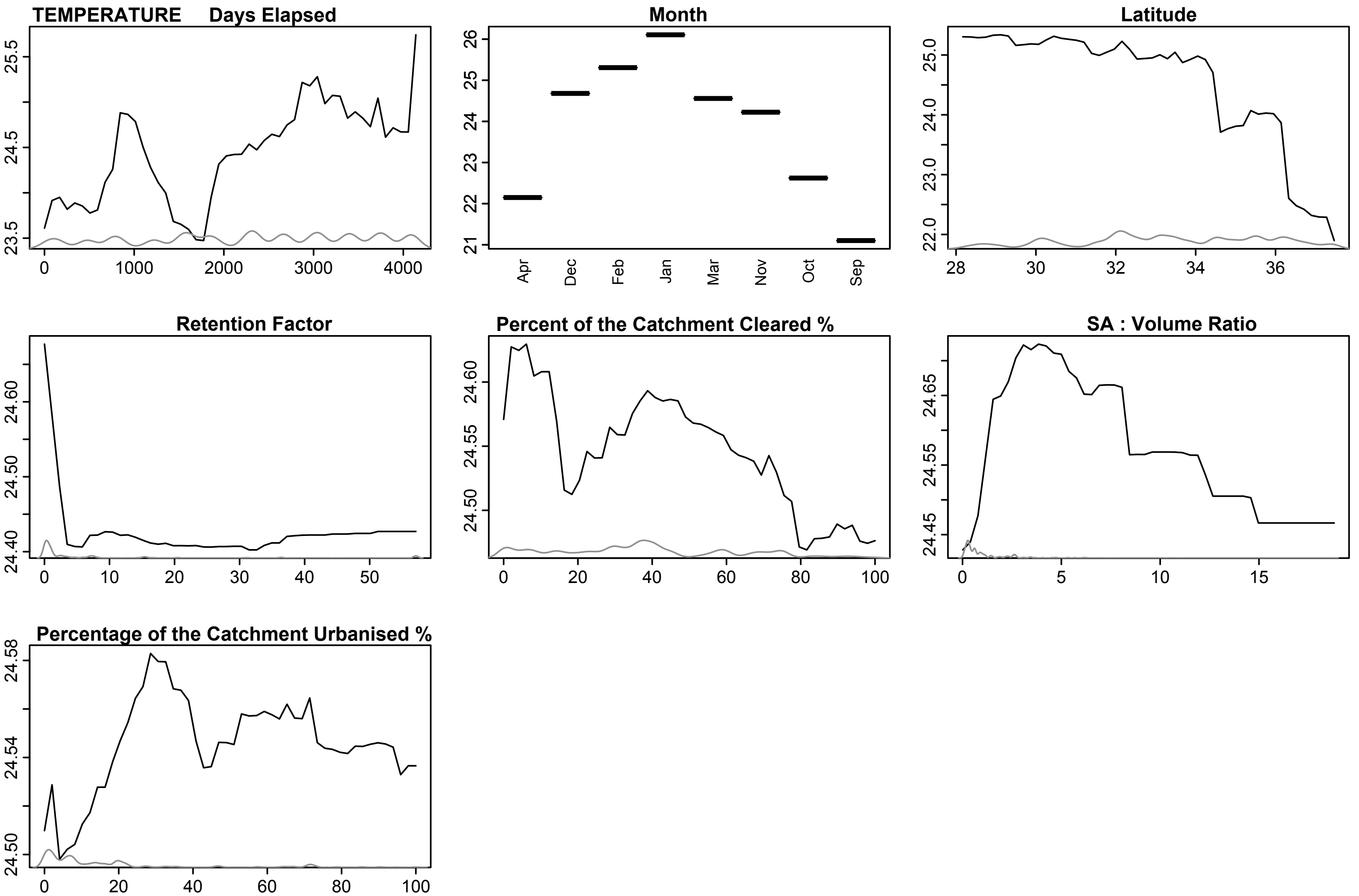

Supplementary Figure 1. Full partial dependence plots for RF models for temperature.
Partial dependence plots for temperature, showing each modelled predictor and response variable.
The small grey line plotted above the x-axis represents the relative density of observations.
Interpretation of plots beyond observed response variables is not advised. Plots were generated using the “plotmo” package.

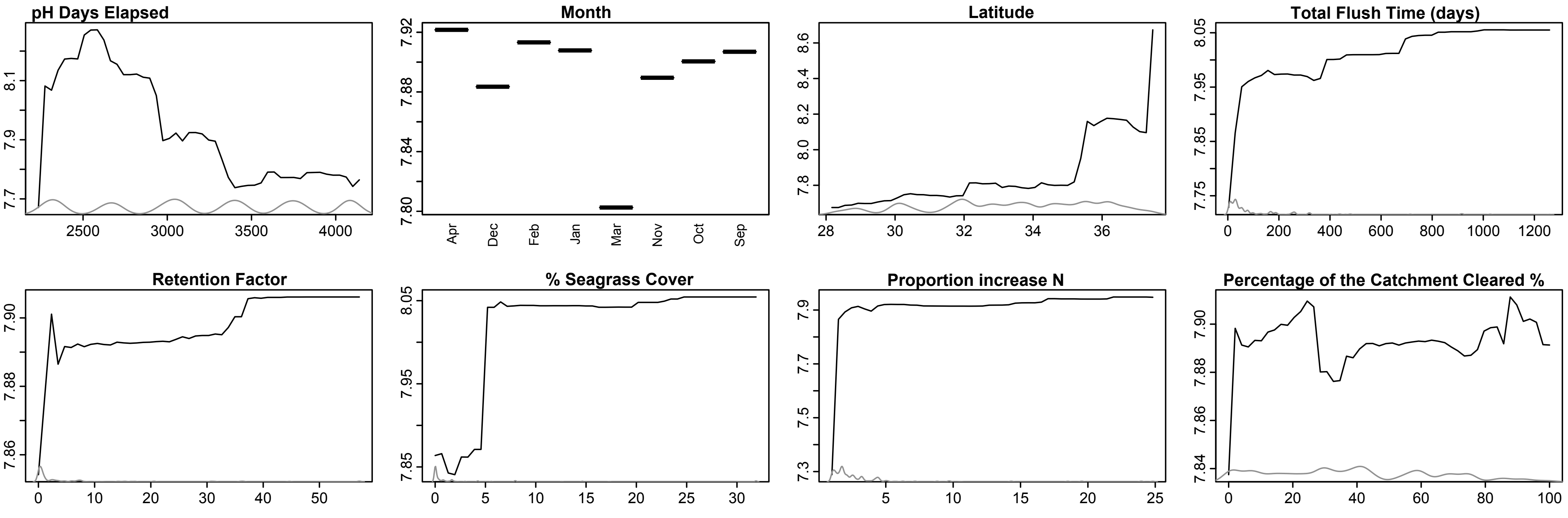

Supplementary Figure 2. Full partial dependence plots for RF models for pH.
Partial dependence plots for pH, showing each modelled predictor and response variable.
The small grey line plotted above the x-axis represents the relative density of observations.
Interpretation of plots beyond observed response variables is not advised. Plots were generated using the “plotmo” package.

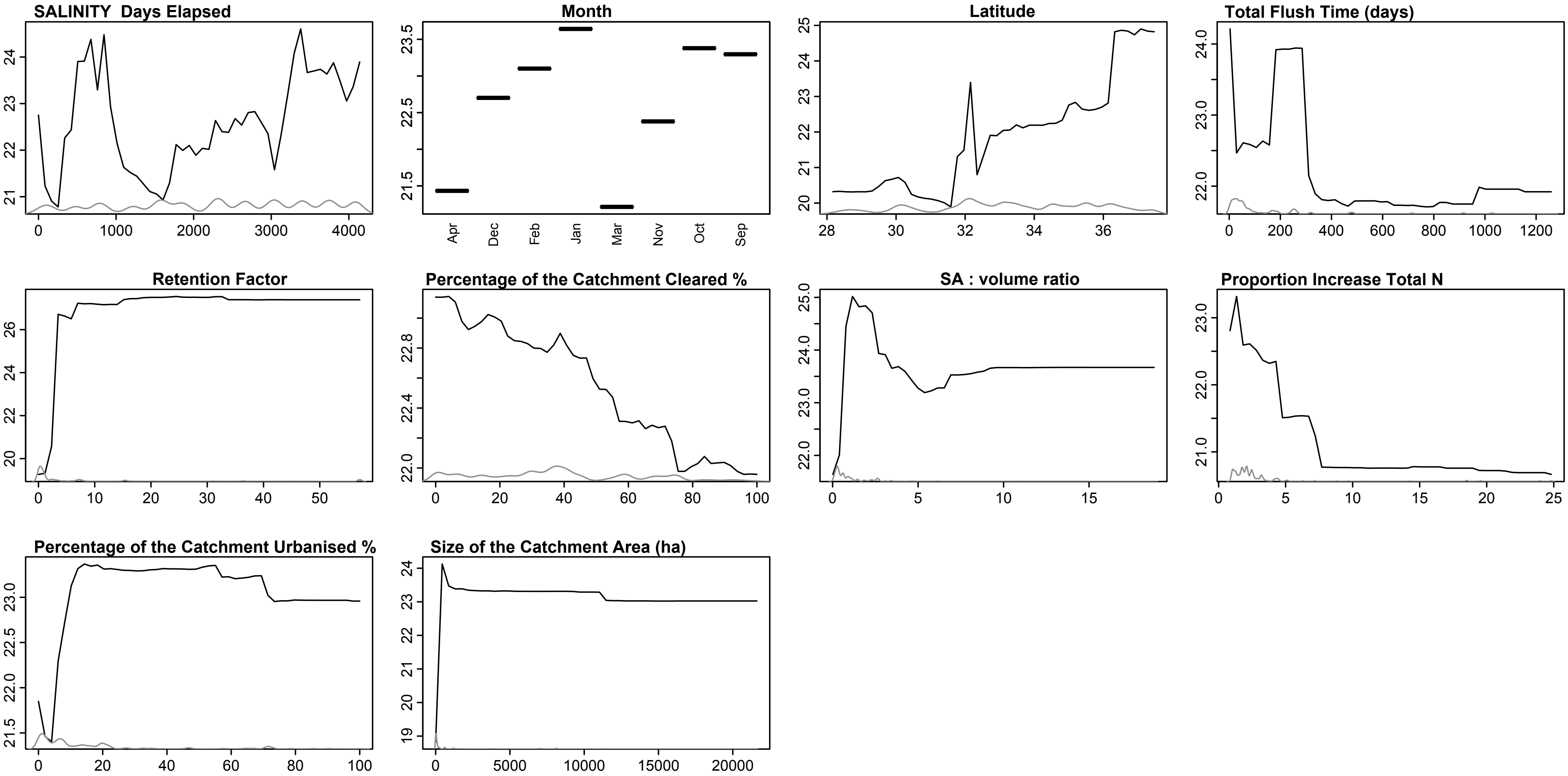

Supplementary Figure 3. Full partial dependence plots for RF models for salinity.
Partial dependence plots for salinity, showing each modelled predictor and response variable.
The small grey line plotted above the x-axis represents the relative density of observations.
Interpretation of plots beyond observed response variables is not advised. Plots were generated using the "plotmo" package.
